# Supplementary material for: Fitting quantum machine learning potentials to experimental free energy data: predicting tautomer ratios in solution
Source: Chem Sci. 2021 Jul 19;12(34):11364–81. doi: 10.1039/d1sc01185e (PMC8409483; doi:10.1039/d1sc01185e)
Supplement: SC-012-D1SC01185E-s001 [file SC-012-D1SC01185E-s001.pdf]

Electronic Supplementary Material (ESI) for Chemical Science.
This journal is © The Royal Society of Chemistry 2021

Fitting quantum machine learning potentials to experimental free energy data: Predicting tautomer ratios in solution

Marcus Wieder* (0000-0003-2631-8415)[1*], Josh Fass* (0000-0003-3719-266X)[1,2], John D. Chodera (0000-0003-0542-119X)[1]

[*]contributed equally to this work; [1]Computational and Systems Biology Program, Sloan Kettering Institute, Memorial Sloan Kettering Cancer Center, New York, NY 10065, USA; [2]Tri-Institutional PhD Program in Computational Biology and Medicine, Weill Cornell Graduate School of Medical Sciences, New York, NY 10065, USA. Current address: Relay Therapeutics, Cambridge, MA 02139, USA

***For correspondence:**
marcus.wieder@choderalab.org (MW)

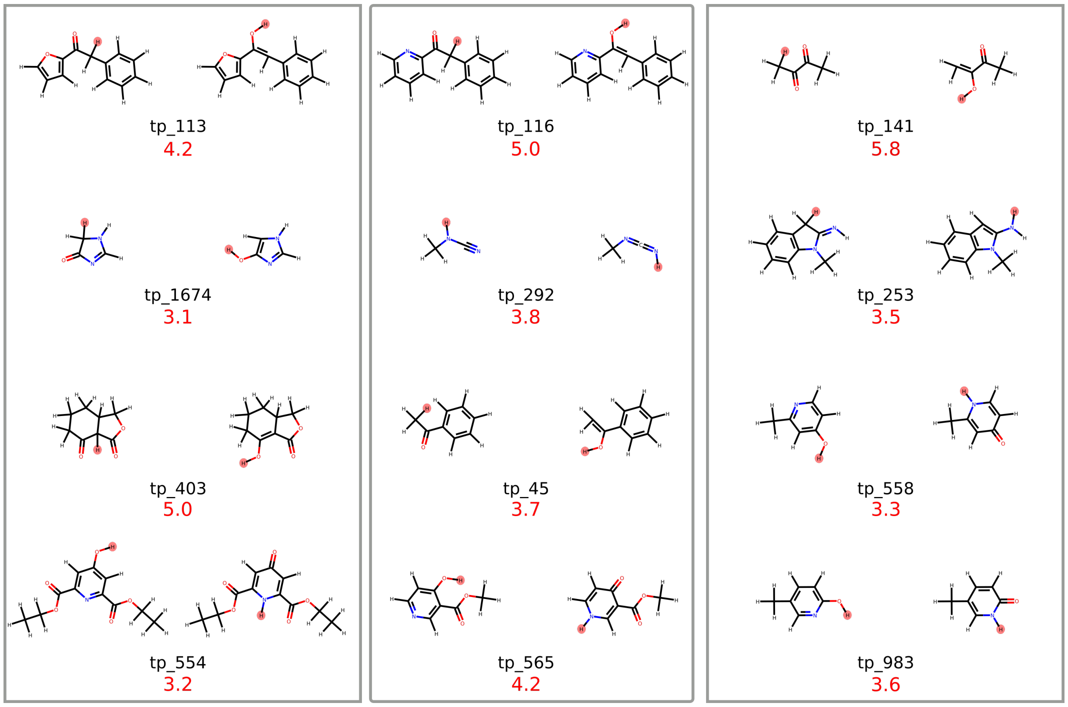

Figure S.I.1. Molecules for which the RRHO approximation introduces an error of more than 3 kcal/mol are shown. Absolute error is shown in red (value in kcal/mol) The hydrogen that changes position is highlighted in red.

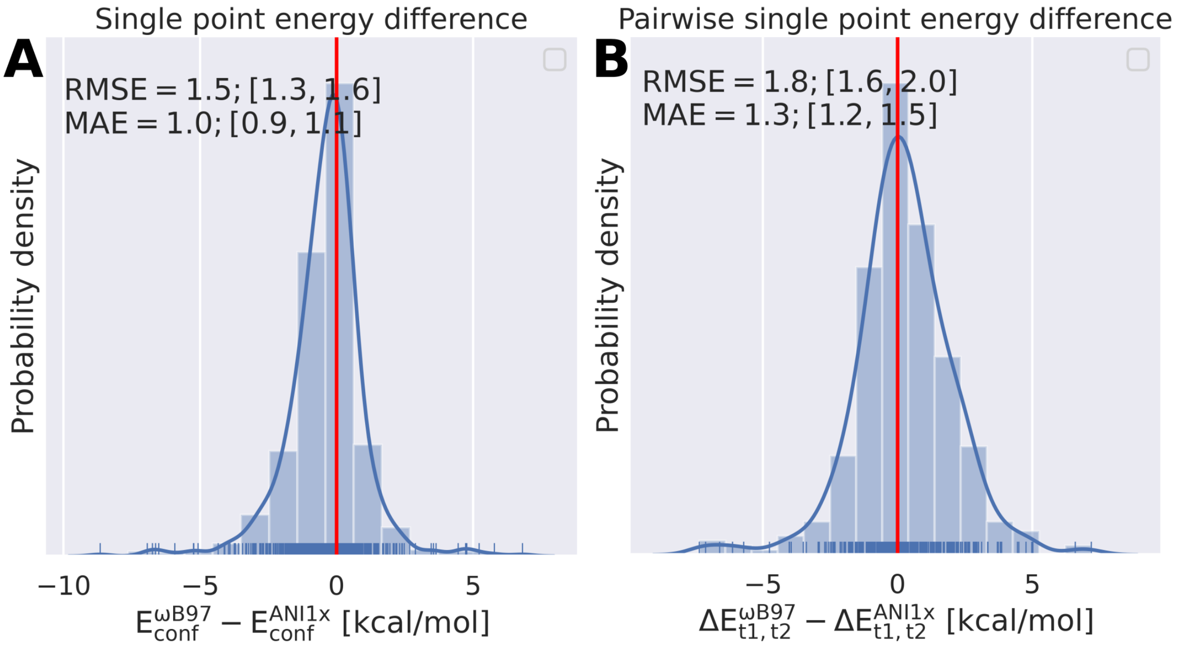

Figure S.I.2. The single point energy deviation between the ANI-1x and its corresponding level of theory is shown for the individual molecules of the tautomer set (A) and for the tautomer pairs (B).

name			ΔG^{exp}_{solv}	ΔG^{calc}_{solv}
tp_1668	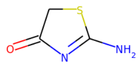	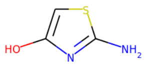	5.5	15.9
tp_1669	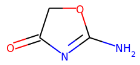	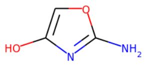	9.5	21.5
tp_1670	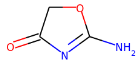	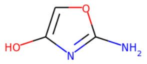	6.8	17.6
tp_1559	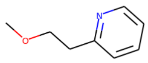	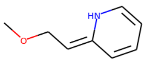	2.7	18.9
tp_331	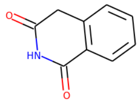	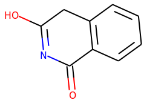	2.2	12.8
tp_853	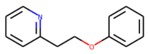	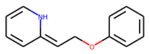	1.4	18.6

Table S.I.1. 5 out of the 6 tautomer pairs with the highest absolute error have common scaffolds. Tautomer pairs with absolute errors above 10 kcal/mol are shown.

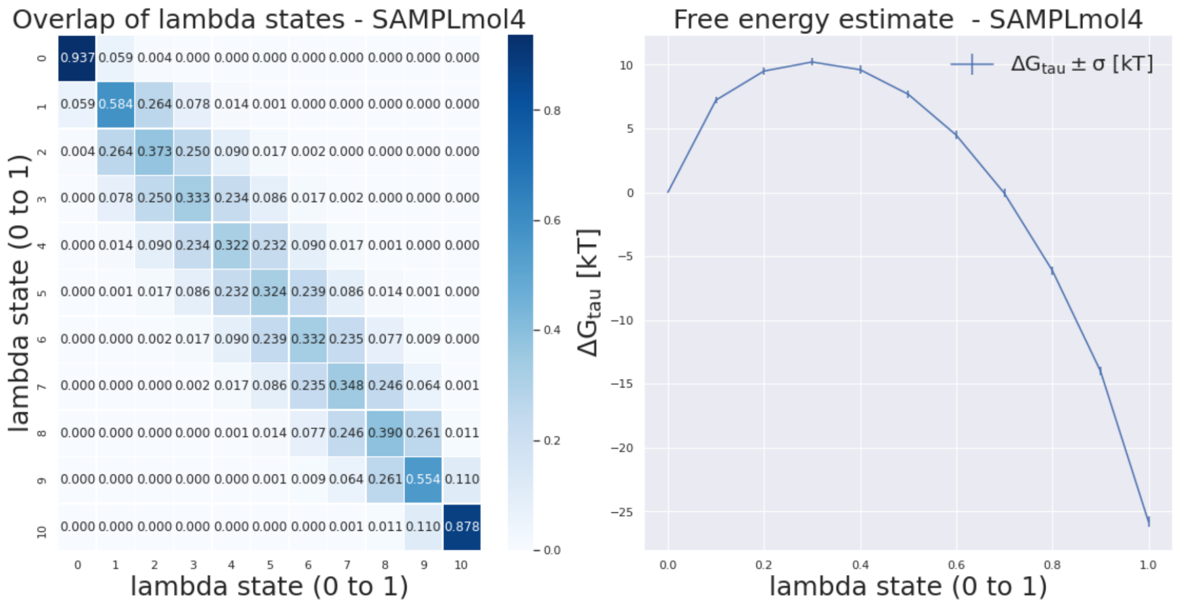

Figure S.I.3. Alchemical free energy overlap and accumulated free energy estimates (with the accumulated free energy uncertainty added) are shown.

tautomer pair	ΔG_{solv}^{calc} [kcal/mol]	ΔG_{vac}^{calc} [kcal/mol]	ΔG_{solv}^{exp} [kcal/mol]
tp_113	4.0 ± 0.2	9.0 ± 0.2	8.0
tp_1000	-0.8 ± 0.2	3.5 ± 0.2	-2.6
tp_1001	-4.5 ± 0.2	-1.8 ± 0.2	-3.7
SAMPLmol4	-6.3 ± 0.2	2.4 ± 0.2	-2.3
SAMPLmol2	-7.6 ± 0.2	-2.7 ± 0.2	-6.1
tp_1072	5.0 ± 0.2	3.4 ± 0.2	0.3
	MAE: 2.8 kcal/mol	MAE: 3.4 kcal/mol	

Table S.I.2. Performing the above described alchemical simulations inside a 16 Å water droplet to model the solvent effects directly shows for 6 selected tautomer pairs an improvement of the mean absolute error by ≈ 0.5 kcal/mol. The alchemical free energy calculation was performed using 11 equidistant lambda states and 200 decorrelated snapshots per lambda state. Samples were drawn from 100 ps simulations.

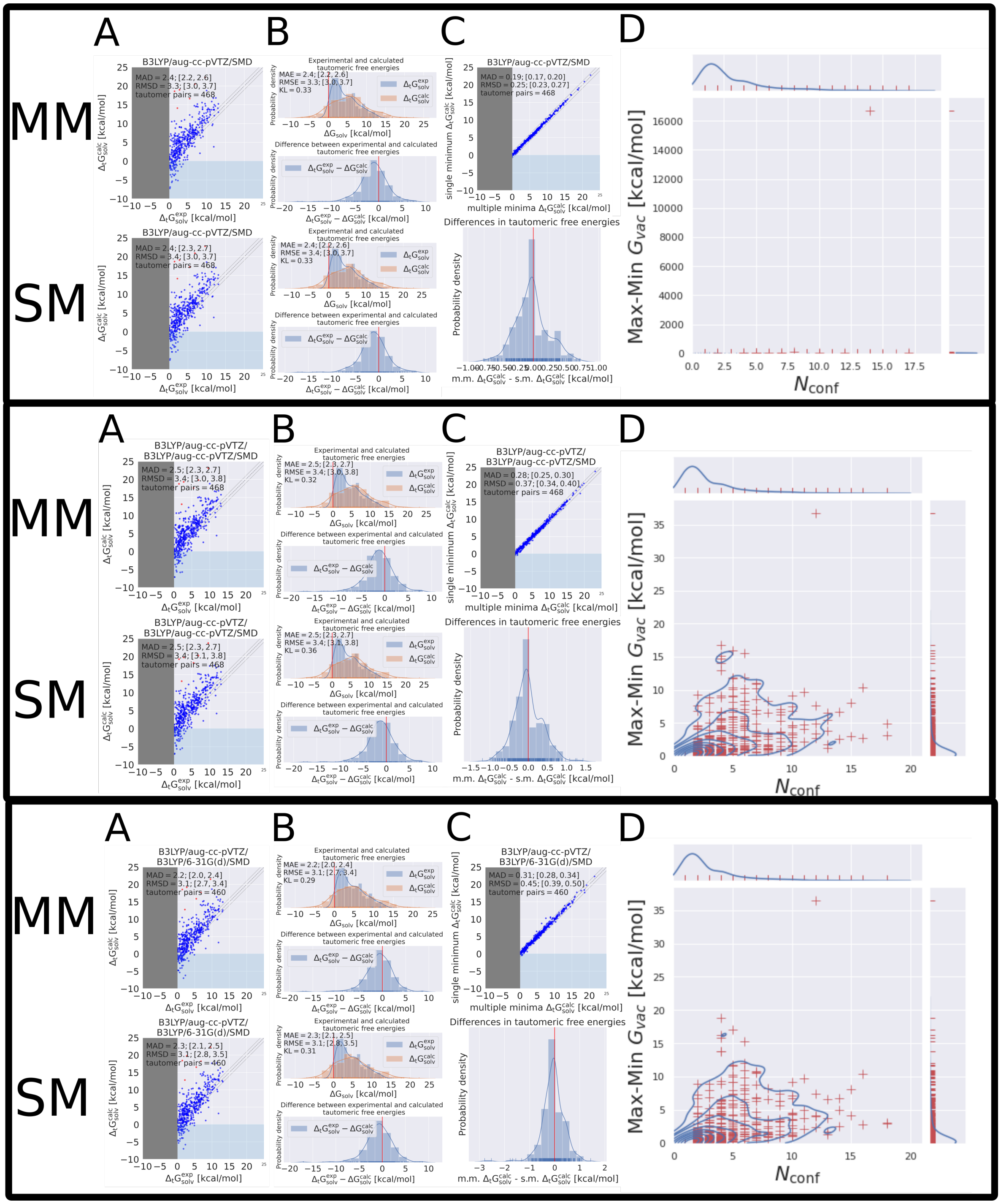

Figure S.I.4. Each of the three blocks show the QM calculations done with different level of theory indicated by the title in the plots in column **A**. In each block the top penal shows the results obtained with multiple minimum conformations, the bottom penal shows the results obtain with a single conformation. Column **D** shows the difference between the multiple minimum and single minimum approach and **E** show the the number of minimum conformations against the difference between the lowest and highest energy for each tautomer. These results were obtained without the additional structure symmetry correction.

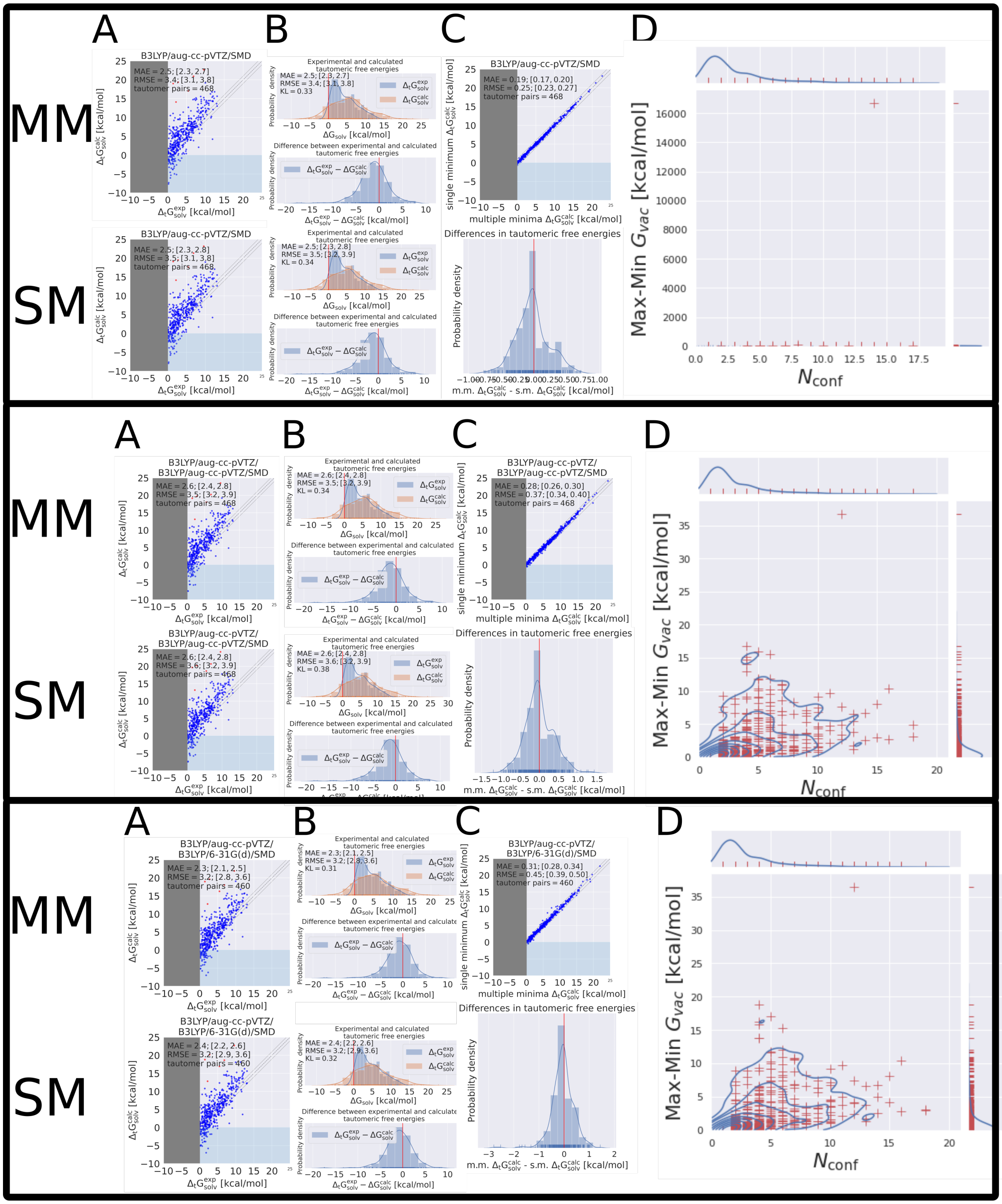

Figure S.I.5. Each of the three blocks show the QM calculations done with different level of theory indicated by the title in the plots in column **A**. In each block the top penal shows the results obtained with multiple minimum conformations, the bottom penal shows the results obtain with a single conformation. Column **D** shows the difference between the multiple minimum and single minimum approach and **E** show the the number of minimum conformations against the difference between the lowest and highest energy for each tautomer. These results were obtained **with** structure symmetry correction.

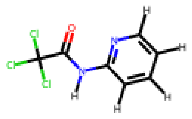

tp_1185-t1

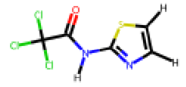

tp_1279-t1

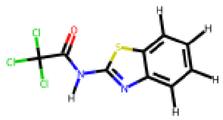

tp_1280-t1

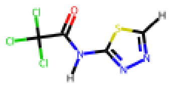

tp_1282-t1

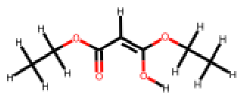

tp_309-t2

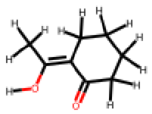

tp_38-t2

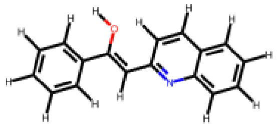

tp_511-t2

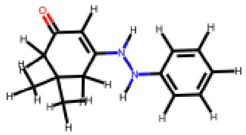

tp_521-t2

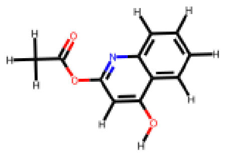

tp_609-t1

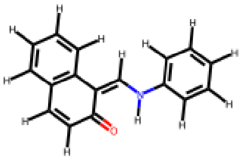

tp_787-t2

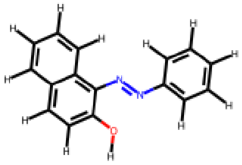

tp_810-t1

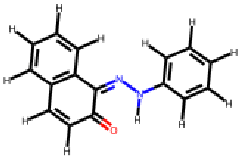

tp_810-t2

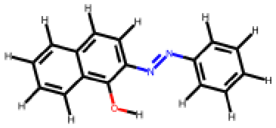

tp_811-t1

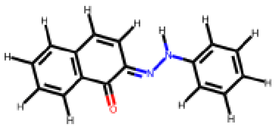

tp_811-t2

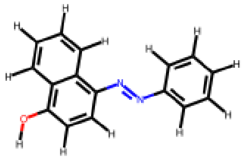

tp_812-t1

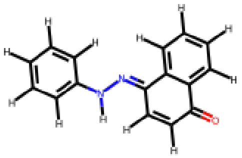

tp_812-t2

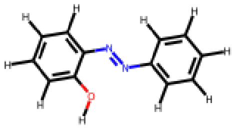

tp_867-t1

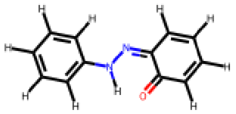

tp_867-t2

Figure S.I.6. Molecules for which the difference between their highest and lowest free energy at a minimum conformation was higher than 10 kcal/mol, calculated with B3LYP/aug-cc-pVTZ. Names are in accordance with Figure S.I.13 and S.I.14.

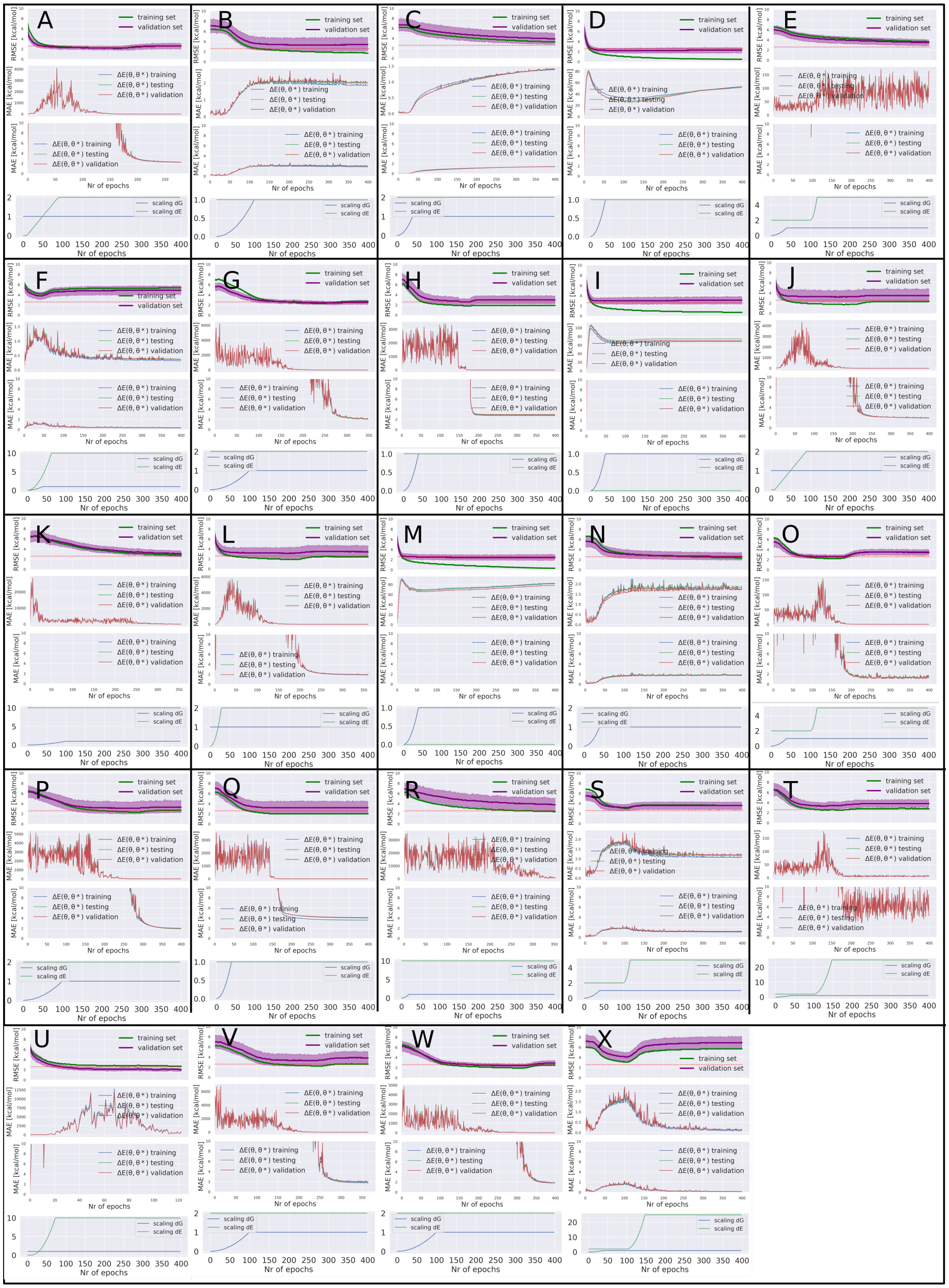

Figure S.I.7. The top panel of the figure shows the RMSE of the training/validation set. The red dotted line indicates the validation set performance of final results shown in Figure 8. The two middle panels show the $\Delta E(\theta, \theta^{*})$ for the coordinate sets in the training (blue), validation (red) and test set (green). The top middle panel shows the full range of the values on the y-axis, while the bottom middle panel is limited to the interval [0,10] kcal/mol. The bottom panel shows the scaling variables used to scale the two terms (free energy and energy deviation) of the molecular loss function. In addition to the scaling variables used in the loss function there were three hyper parameters controlling the performance of the optimizer: the learning rate (LR) for the SGD and AdamW optimizer (optimizing the bias and the weight of the neural net) and the weight decay. The following list contains the LR for the training runs shown, if weight decay or a learning rate reduction method was used it is explicitly mentioned. **A, H, J, K, L, M, R, U, V, W**: AdamW: LR of 1e-4, SGD: LR of 1e-4. **B** : LR AdamW: 1e-4, LR SGD: 1e-9. **F, I, N, X, S**: LR AdamW: 1e-4, LR SGD: 0. **D**: LR AdamW 1e-5, LR SGD: 0. **O**: LR AdamW 1e-4, LR SGD: 1e-6. **E** : LR AdamW 1e-5, LR SGD: 1e-6. **G** : LR AdamW 1e-4, LR SGD: 1e-4, weight decay: 1e-05. **Q** : LR AdamW 1e-4, LR SGD: 1e-4, with LRReduction on Plateau for AdamW. **T** : LR AdamW 1e-4, LR SGD: 1e-6, weight decay: 1e-9.

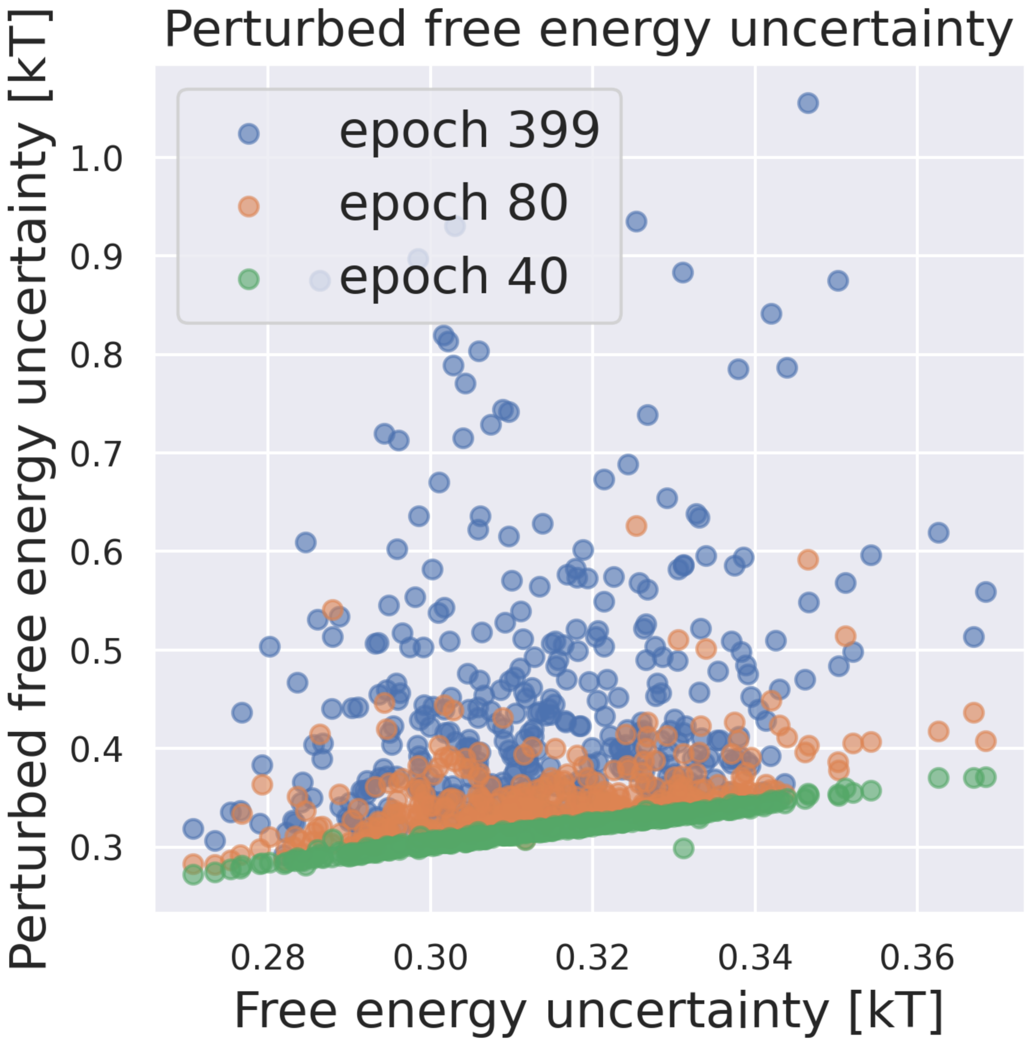

Figure S.I.8. Perturbed free energy uncertainty increases with the number of training epochs. The free energy estimate uncertainty was calculated using the MBAR implementation in pyMBAR.

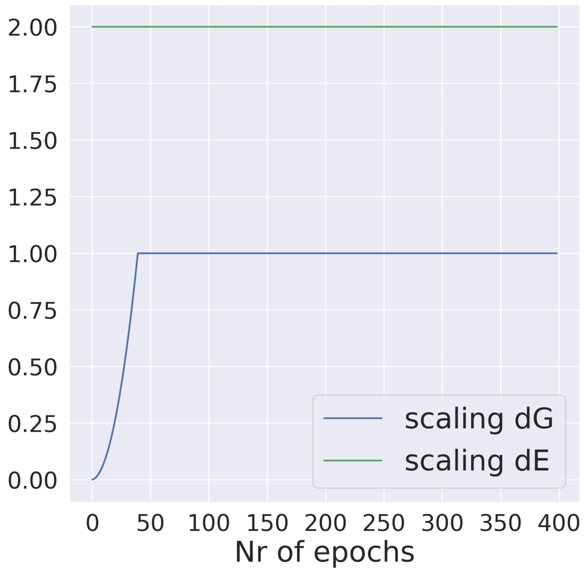

Figure S.I.9. The scaling factors for f(epoch) and g(epoch) used in the molecular loss function in the reported results in 8.

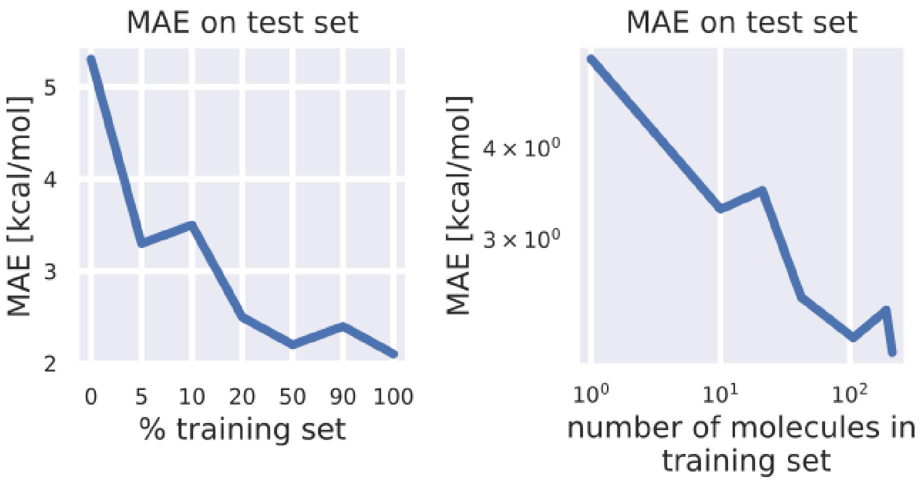

Figure S.I.10. The learning curve shows the performance of the optimized parameter set on a test set resulting from different retraining runs performed with increasing percentage of the training set. Scaling factors and learning rate were chosen as in Figure 8. The MAE was calculated with the parameter set with the best performance on the validation set. The training and validation set was kept constant, molecules from the training set were randomly selected.

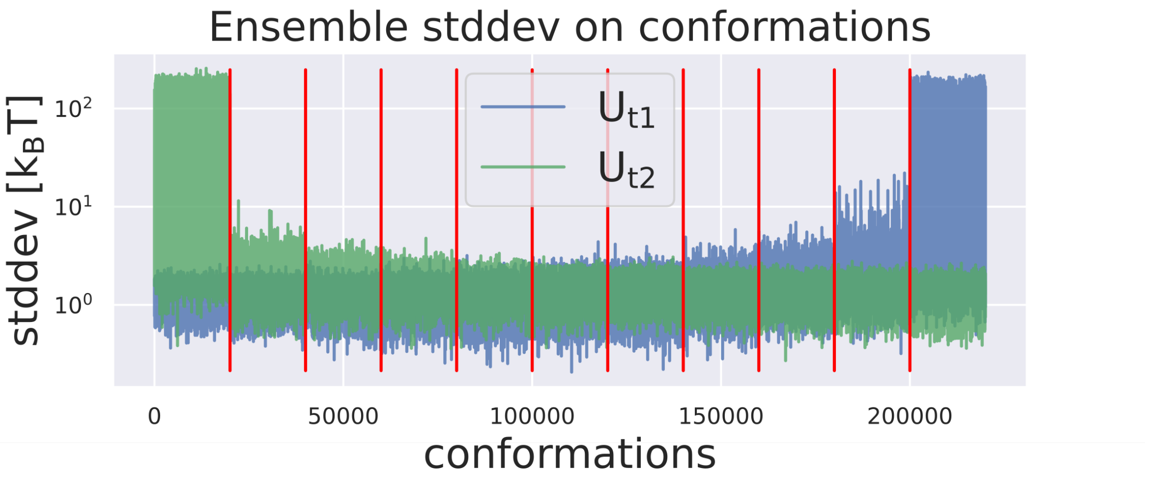

Figure S.I.11. The neural net ensemble standard deviation is plotted for the potential energy calculation with the end-point potential energy functions (U_{t1} and U_{t2}) on the conformations obtained along the alchemical path for the 11 λ states, starting with conformations from $\lambda = 0$. The red lines indicate the different states along the alchemical path starting at the left end with $\lambda = 0$ and ending at $\lambda = 1$ The slow increase of the standard deviation along the alchemical path indicates that ANI-1ccx is well suited to efficiently perform alchemical free energy calculations for tautomer pairs and the QML has confidence in the potential energy values even at $\lambda = 0.5$. The high standard deviation for U_{t1} on samples generated at $\lambda = 1$ and vice versa are to be expected since the dummy hydrogen of U_{t2} at $\lambda = 1$ is moving freely inside a spherical restraint; but this hydrogen is a real atom for U_{t1}.

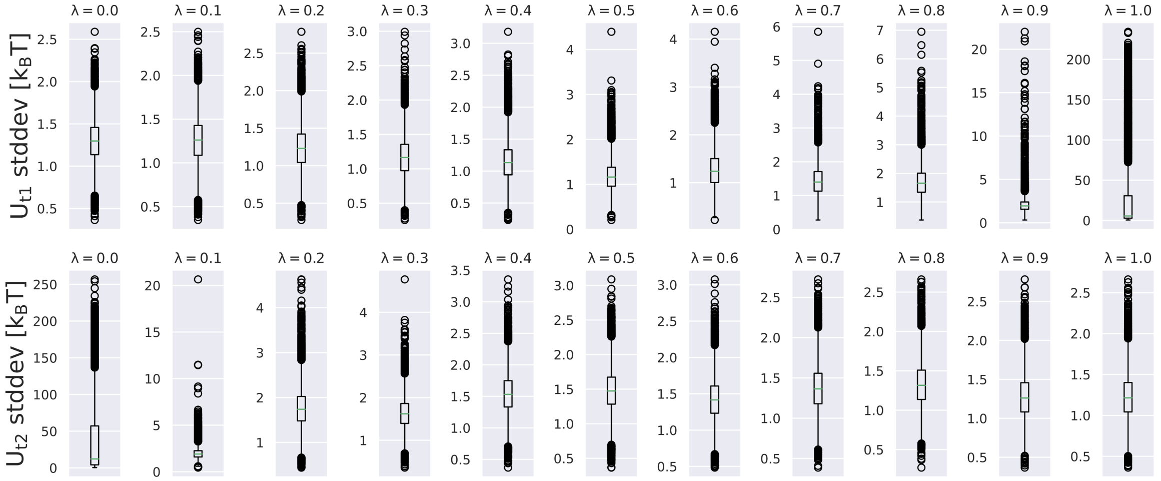

Figure S.I.12. The neural net ensemble standard deviation is plotted for the potential energy calculation with the end-point potential energy functions (U_{t1} and U_{t2}) on the conformations obtained along the alchemical path for the 11 λ states.

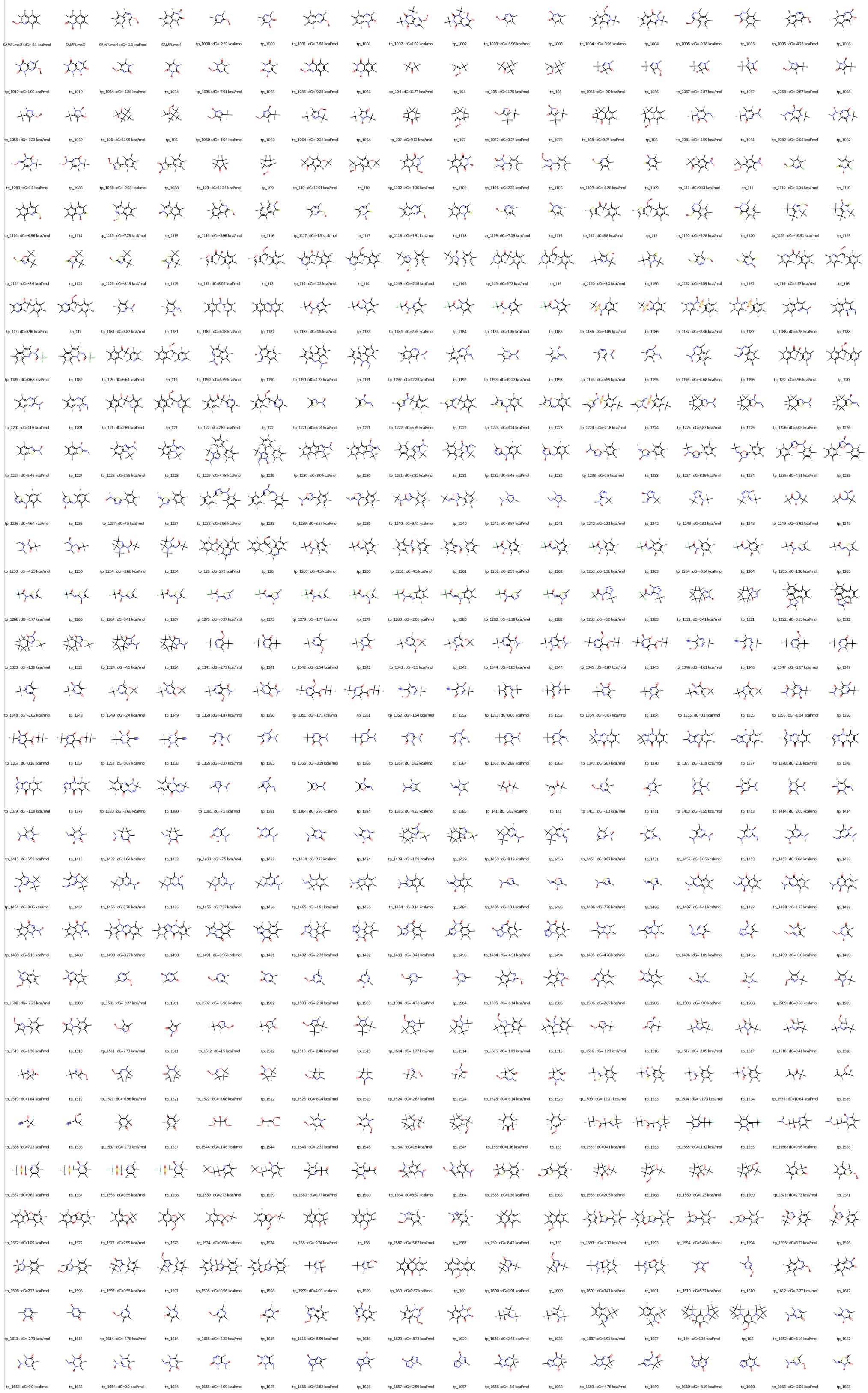

Figure S.I.13. The full tautomer set is shown (part 1). The hydrogen that is moved in the reaction is highlighted in red, ΔG indicates the experimental free energy difference in solution.

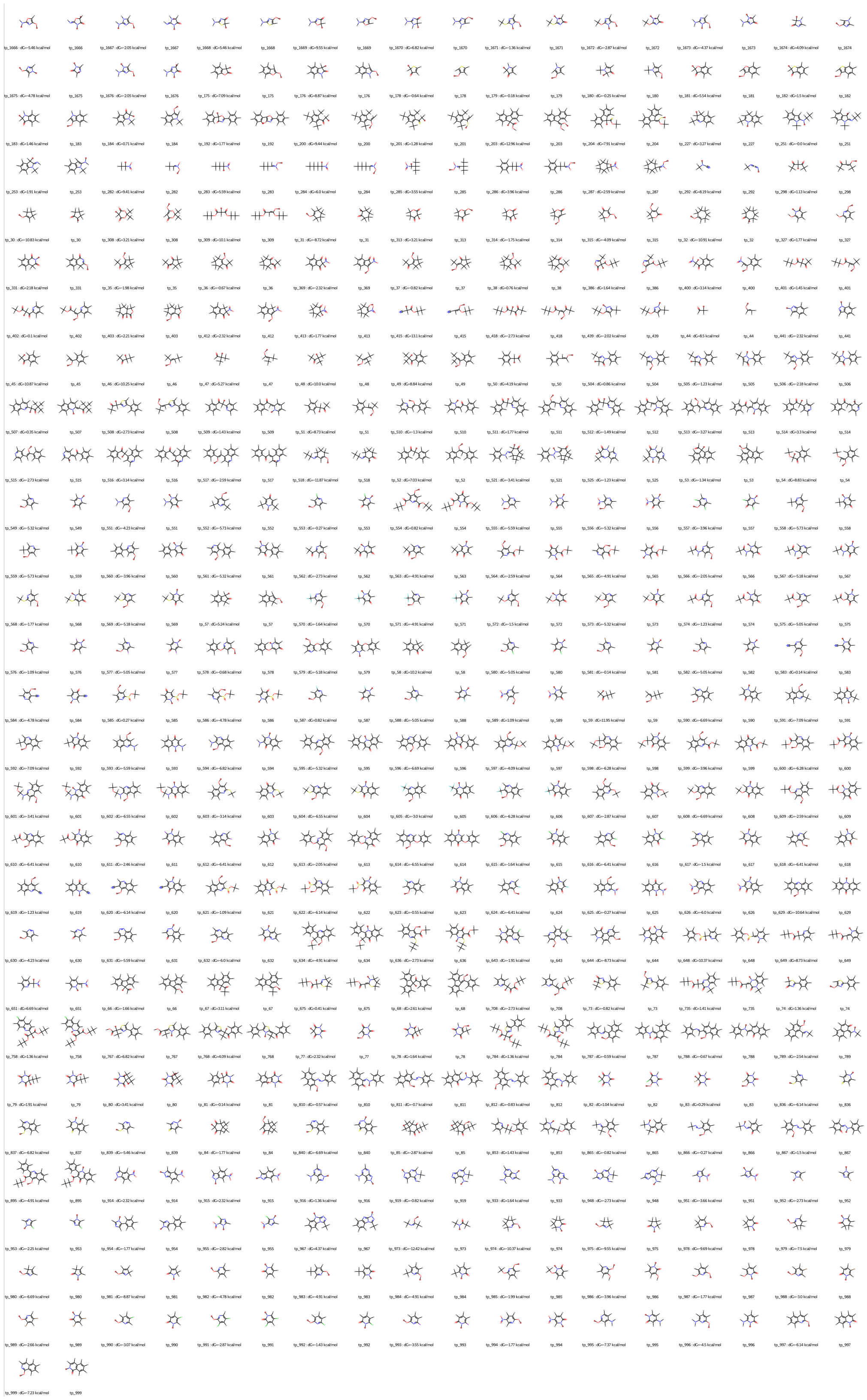

Figure S.I.14. The full tautomer set is shown (part 2). The hydrogen that is moved in the reaction is highlighted in red, ΔG indicates the experimental free energy difference in solution.
